# Functional and Morphological Cardiac Magnetic Resonance Imaging of Mice Using a Cryogenic Quadrature Radiofrequency Coil

**DOI:** 10.1371/journal.pone.0042383

**Published:** 2012-08-01

**Authors:** Babette Wagenhaus, Andreas Pohlmann, Matthias Alexander Dieringer, Antje Els, Helmar Waiczies, Sonia Waiczies, Jeanette Schulz-Menger, Thoralf Niendorf

**Affiliations:** 1 Berlin Ultrahigh Field Facility (B.U.F.F.), Max-Delbrück Center for Molecular Medicine, Berlin, Germany; 2 Charité Working Group Cardiac MRI, Experimental and Clinical Research Center and HELIOS Clinics, Berlin, Germany; 3 Experimental and Clinical Research Center, a joint cooperation between the Charité Medical Faculty and the Max-Delbrück Center for Molecular Medicine, Berlin, Germany; Osaka University Graduate School of Medicine, Japan

## Abstract

Cardiac morphology and function assessment by magnetic resonance imaging is of increasing interest for a variety of mouse models in pre-clinical cardiac research, such as myocardial infarction models or myocardial injury/remodeling in genetically or pharmacologically induced hypertension. Signal-to-noise ratio (SNR) constraints, however, limit image quality and blood myocardium delineation, which crucially depend on high spatial resolution. Significant gains in SNR with a cryogenically cooled RF probe have been shown for mouse brain MRI, yet the potential of applying cryogenic RF coils for cardiac MR (CMR) in mice is, as of yet, untapped. This study examines the feasibility and potential benefits of CMR in mice employing a 400 MHz cryogenic RF surface coil, compared with a conventional mouse heart coil array operating at room temperature. The cryogenic RF coil affords SNR gains of 3.0 to 5.0 versus the conventional approach and hence enables an enhanced spatial resolution. This markedly improved image quality – by better deliniation of myocardial borders and enhanced depiction of papillary muscles and trabeculae – and facilitated a more accurate cardiac chamber quantification, due to reduced intraobserver variability. In summary the use of a cryogenically cooled RF probe represents a valuable means of enhancing the capabilities of CMR of mice.

## Introduction

Genetically modified animal models have evolved into protagonists in cardiac research. Alongside the established surgically and pharmacologically induced animal models, they are valuable for studying the pathogenesis of cardiovascular diseases. Adequate characterization, rapid phenotyping, assessment of gender effects, evaluation of novel therapeutics and long-term follow-up studies are still challenging. To this end there is an ever growing need for non-invasive *in vivo* imaging that (i) provides excellent spatial and temporal resolution (a prerequisite for cardiovascular research), (ii) has a high reproducibility and (iii) is suitable for longitudinal studies. Cardiovascular magnetic resonance (CMR) and echocardiography are widely used in clinical and preclinical studies, with CMR being accepted as the more accurate modality. Cardiac morphology and function assessment is the workhorse of CMR and is of increasing interest for a variety of mouse models in pre-clinical cardiac research, such as myocardial infarction models or myocardial injury/remodeling in genetically or pharmacologically induced hypertension.

The small size of the mouse heart and its rapid motion with typical heart rates of 400 to 600 beats per minute pose substantial challenging demands on CMR in mice. Together with signal-to-noise ratio (SNR) limitations, these challenges bear the potential to deteriorate image quality severely. Cardiac morphology and function assessment requires imaging techniques that provide excellent delineation of myocardial borders - hence the need for high ventricular blood to myocardium contrast -, full coverage of the cardiac cycle with high temporal resolution, but also coverage of the entire heart with high spatial resolution to facilitate reliable segmentation of the endo- and epicardial borders.

Realizing these competing constraints assessment of cardiac morphology, cardiac chamber quantification and cardiac function assessment require imaging protocols and hardware that are tailored for CMR. The hardware used in current experimental CMR of mice affords images with a typical in-plane spatial resolution of 100–200 µm, heart coverage of 6–13 slices of 1.0 mm thickness and measurement of 10–20 cardiac phases [1], [2], [3], [4], [5], [6], [7], [8], [9], [10]. Although these studies provide acceptable image quality for the quantitative assessment of the left ventricle (LV), assessment of the right ventricle (RV) remains challenging due to the limited image quality of the RV. RV assessment is of increasing interest since remodelling and myocardial injury are not restricted to the left ventricle [11] and the RV is a primary target of diseases including RV dysfunction or RV hypertrophy as a result of pulmonary hypertension or RV-thinning in case of dysplasia, such as arrhythmogenic right ventricular cardiomyopathy (ARVC) [12]. For preclinical research of these diseases numerous mouse models are available, such as pulmonary arterial constriction [13] (for the RV aspects of pulmonary hypertension) or plakoglobin deficient mice [14] (for ARVC). For all these reasons it is conceptually appealing to improve image quality for CMR in mice. To meet this goal enhancements in the spatial resolution are essential, which in turn built on SNR improvements.

Strategies for improving SNR are i) signal averaging, which comes at the expense of prolonged measurement times, ii) moving to higher magnetic field strengths, which are associated with practical impediments, such as increased magnetic field inhomogeneities and RF power deposition, or iii) perfecting radio frequency (RF) coil technology, which holds the promise to offer significant gains in SNR. RF coils tailored for CMR are dedicated birdcage volume coils or surface coil arrays with the geometry adjusted to the size of a mice (25–35 mm inner diameter or 15–26 mm side length respectively) [2], [4], [5], [6], [7], [8], [10]. An emerging RF coil technology for MRI of small rodents is the cryo-cooling of RF coils, which reduces the electronic noise. Such cryogenic RF probes are widely used in vertical NMR spectroscopy systems, but have only recently become available for horizontal small bore MR imaging systems. Significant gains in SNR with a cryogenic probe have been shown for MRI of the mouse brain [15], [16], [17], [18]. The application of cryogenic RF coils for CMR in mice has, as of yet, untapped potential. No reports that exploit cryogenic RF coil technology for CMR in mice have been published yet.

Realizing this opportunity, this study examines the feasibility of cardiac MRI in mice employing a 400 MHz cryogenic transceive RF surface coil and demonstrates its suitability for high spatial resolution functional and structural CMR in mice. Image quality, SNR, and cardiac function are assessed. For comparison a conventional mouse heart receive-only coil array operating at room temperature is used.

## Materials and Methods

### Ethics Statement

Animal experiments were carried out in accordance with the guidelines provided and approved by the Animal Welfare Department of the *Landesamt für Gesundheit und Soziales* Berlin (Berlin State Office of Health and Social Affairs, Permit Number: G0019/12). All imaging experiments were performed under isoflurane anesthesia, and all efforts were made to minimize suffering.

### MRI System and Coil Setup

All imaging experiments were carried out on a 9.4T small animal MR system (Biospec 94/20, Bruker Biospin, Ettlingen, Germany) operating at 400 MHz. Two RF coil set-ups were used:

- conventional linear polarized birdcage resonator (Bruker Biospin, Ettlingen, Germany; inner diameter of 72 mm) for transmission in conjunction with a curved four channel receive only mouse cardiac coil array (Bruker Biospin, Ettlingen, Germany) at room temperature (RT) and
- a cryogenic transceive quadrature RF surface coil (CryoProbe, CP, Bruker Biospin, Ettlingen, Germany) of similar coil geometry as the RT surface coil (overlapping coils bent to a cylinder surface; inner diameter: 20 mm, length: 85 mm) operating at around 30 K (preamplifiers at 77 K) cooled by a closed-cycle refrigeration system.

The CryoProbe’s surface (which is in direct contact with the animal) is equipped with a temperature sensor and can be temperature regulated using a resistive heater unit (Bruker, BioSpin AG, Fällanden, Switzerland). This approach was used to maintain the surface temperature of the Cryoprobe at 37°C.

### Animal Preparation

3-month-old C57BL/6N mice were obtained from a commercial breeder (Charles River, Sulzfeld, Germany) and kept under controlled conditions for temperature, humidity and light, with chow and water ad libitum. Mice were anesthetized in a warmed anesthetic chamber using 2.5% isoflurane in an oxygen/air mixture (2∶1) with a flow rate of 750 ml/min for induction and then maintained at 1.5%. When using the CryoProbe the forepaws of the mouse were held in a caudal position by adhesive tape in order to keep the distance between mouse chest and coil elements as small as possible. The mouse was placed supine on the animal bed of the CryoProbe, in contrast to the prone position (with cranial forepaw placement) required for use of the RT-coil.

A body temperature of 37°C was maintained by warming the animal beds using a circulating heated water system (Thermo Haake GmbH, Karlsruhe, Germany) and monitoring by a rectal temperature probe throughout the measurements. Respiratory signals were continuously monitored using a commercial monitoring and gating system (SA Instruments, Inc., New York, USA) and kept between 50–70 respiration cycles per minute by regulating the isoflurane dose. Ten mice were imaged twice, once with the RT-coil and once with the CryoProbe in an interval of two weeks. The first and second MRI was performed at the same time of the day to avoid physiological variations due to circadian rhythm.

### B_1_ Field of the CryoProbe in vivo

Relative maps of the transmit component of the RF electro-magnetic field (B_1_^+^) were calculated from pulse oximetry triggered FLASH acquisitions (α = 30°/60°, TE/TR  = 1.4/5000 ms, BW = 75 kHz, slice thickness  = 2 mm, spatial resolution: 0.23×0.71×2 mm^3^ interpolated to 0.12×0.23×2 mm^3^, averages  = 2, partially parallel imaging (PPI) acceleration  = 2, acquisition time per angle  = 7 min) of standard short axis and four chamber views of the mice heart using the double angle method [19], [20].

### In vivo Coil Comparison

To obtain a stack of cardiac short axis (SAX) views covering the whole heart, ten slices were consecutively acquired using a self-gated [4] bright-blood cine fast low angle shot technique (IntraGate-FLASH, slice thickness  = 0.8 mm). For each coil two imaging protocols were conducted using:

- conventional spatial resolution protocol (spatial resolution  = 156×234×800 µm^3^, TE/TR = 1.4/8.5 ms, α = 15°, BW = 75 kHz, NR = 100, cardiac frames  = 20, TA ∼2 min) and
- high spatial resolution protocol (spatial resolution  = 69×115×800 µm^3^, TE/TR  = 1.3/8.5 ms, α = 20°, BW = 75 kHz, NR = 170, cardiac frames  = 20, TA ∼4.5 min).

For both protocols the navigator parameters used for motion tracking and cardiac gating were: FA = 2°, BW = 2740 Hz, thickness  = 2 mm in oblique slice location. The navigator slice was always aligned with the long axis of the left ventricle and crossed the apex and the center of the mitral valve. Since volume resonators and surface coils have different B_1_^+^ efficiency and characteristics, the RF power adjustment slice (thickness  = 2 mm) was adapted to the respective coil configuration. For the RT-coil the adjustment slice was positioned in axial orientation whereas the adjustment slice for the CryoProbe experiments was placed in the coronal orientation through the center of the heart.

For SNR comparison between both imaging protocols the mean SNR of left ventricular myocardium was estimated for a midventricular slice. The high-resolution protocol was used for in-depth SNR analysis and cardiac function assessment. SNR values were obtained by determining signal intensities from regions of interest (ROI) positioned in an end diastolic short axis view on the level of the papillary muscles using a six segment model [21]. Mean myocardium SNR values were estimated for all slices covering the heart from the base to the apex. Noise was given by the standard deviation of the signal obtained from a ROI placed in the background (air) anterior to the thorax on the midventricular level. SNR was estimated by dividing mean signal intensity of the left ventricular myocardium by the noise. Cardiac function assessment was performed twice by an experienced observer in a blinded manner using CMR42 (Circle CVI, Calgary, Canada). For this purpose endo- and epicardial borders were contoured in end-systole and end-diastole using a stack of short axis cine views. Stroke volumes (SV), end-diastolic volumes (EDV), end-systolic volumes (ESV), ejection fractions (EF), and myocardial masses (EDM) were calculated for both left and right ventricle. Intraobserver and interobserver variability for the EDV, ESV and EDM of the left and right ventricle was evaluated using Bland-Altman analysis.

To further put the SNR gain inherent to the CryoProbe to use CINE images were acquired with an exquisite spatial resolution of (43×138×300) µm^3^ using short axis views (TE/TR = 1.6/12.5 ms, α = 22°, partial Fourier acceleration  = 2, BW = 119 kHz, NR = 340, cardiac frames  = 20, TA ∼19 min, called *ultra-high resolution* in the following).

## Results

### Transmit Component RF Field (B1^+^) in vivo

**Figure 1:**
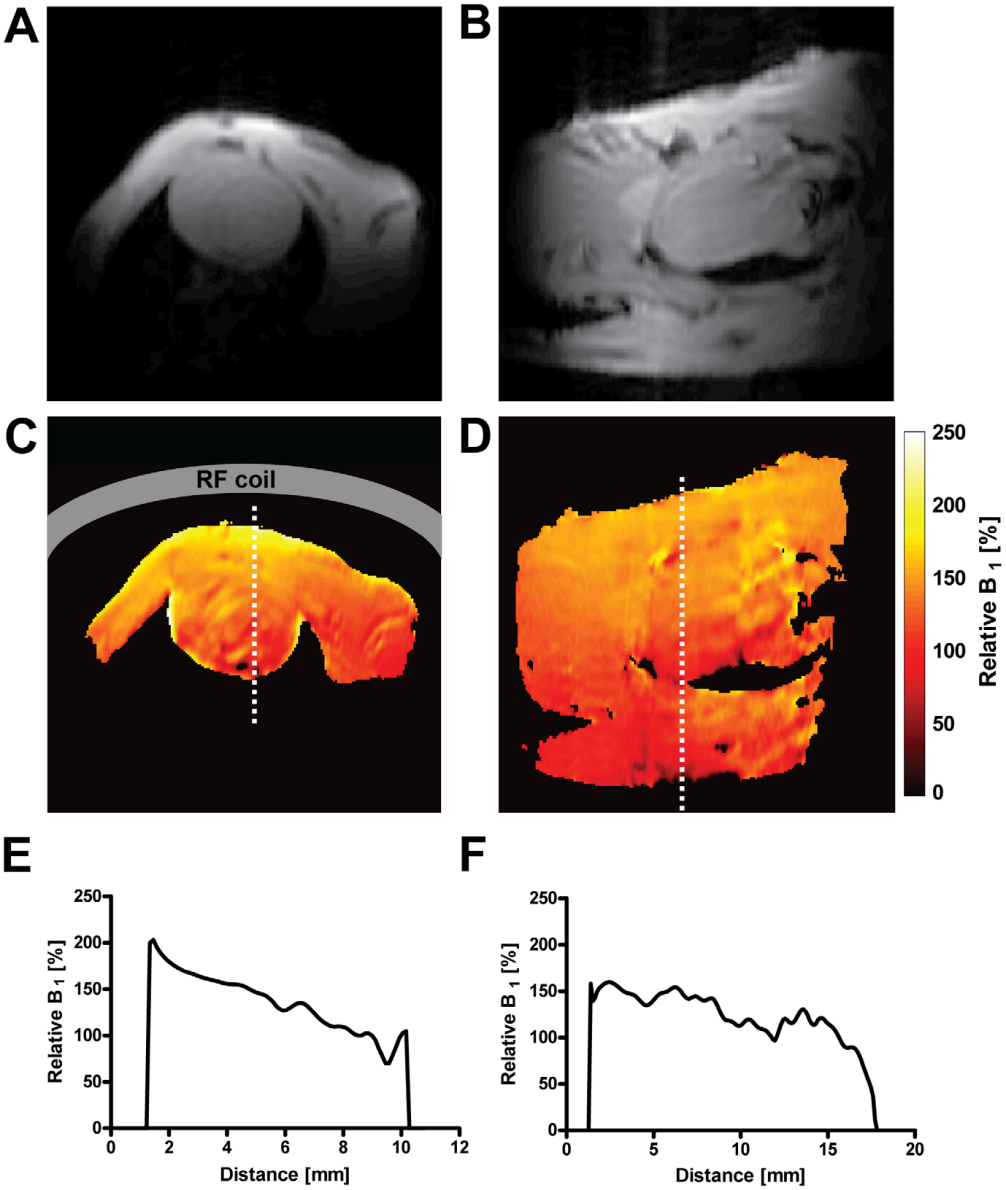
Relative B_1_^+^-maps of a short axis view (c) and a four chamber view (d) using the CryoProbe, with the corresponding MR images (a and b respectively) of identical image geometry. The coil is positioned on the upper side (anterior), schematically shown as a gray bar in (c). Compared to the B_1_^+^-maps (c,d), B_1_^+^-profile plots (e,f) allow for a more precise presentation of the spatial B_1_^+^-variation. These plots show the B_1_^+^ profile along a line crossing the heart (dotted line in (c) and (d) respectively). The B_1_^+^ decrease from anterior to posterior is approximately linear (c).

Relative B_1_^+^-maps of a four chamber view and a short axis view obtained for the CryoProbe are shown in *Figure 1*. A linear B_1_^+^ profile was found for a midventricular short axis view. A change in realtive B_1_^+^ of approximately 65% was observed in the coil sensitivity profile ranging from the anterior chest wall to the inferior myocardium. The long axis view revealed a rather constant B_1_^+^ distribution due to an almost parallel alignment of the slice with the coils sensitivity profile. B_1_^+^ values in the region of the myocardium were found to be above 100%.

### In vivo Imaging of Mice Heart

**Figure 2:**
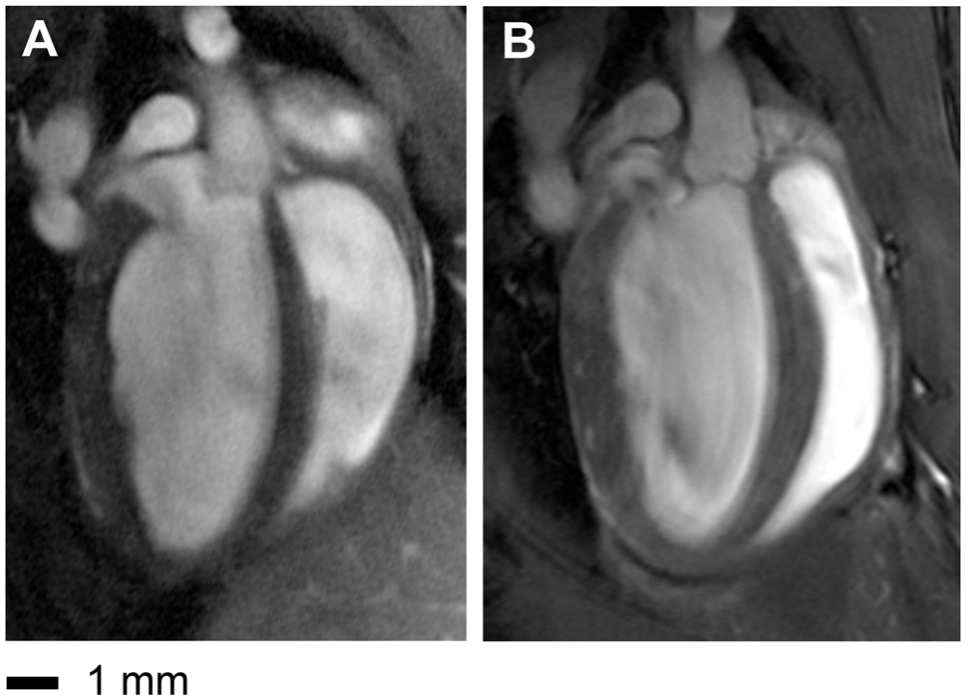
Comparison between high resolution (69×115×800 µm^3^) images of a four chamber view acquired with the birdcage resonator in conjunction with a four channel mouse receive only cardiac surface coil array at room temperature (a) and the CryoProbe (b). Contrary to the room temperature coil image, the CryoProbe image accurately delineates the aortic valve, the left ventricular outflow tract including aortic vessel wall, as well as the lateral right ventricle blood myocardium border especially at the level of the apex.

**Figure 3:**
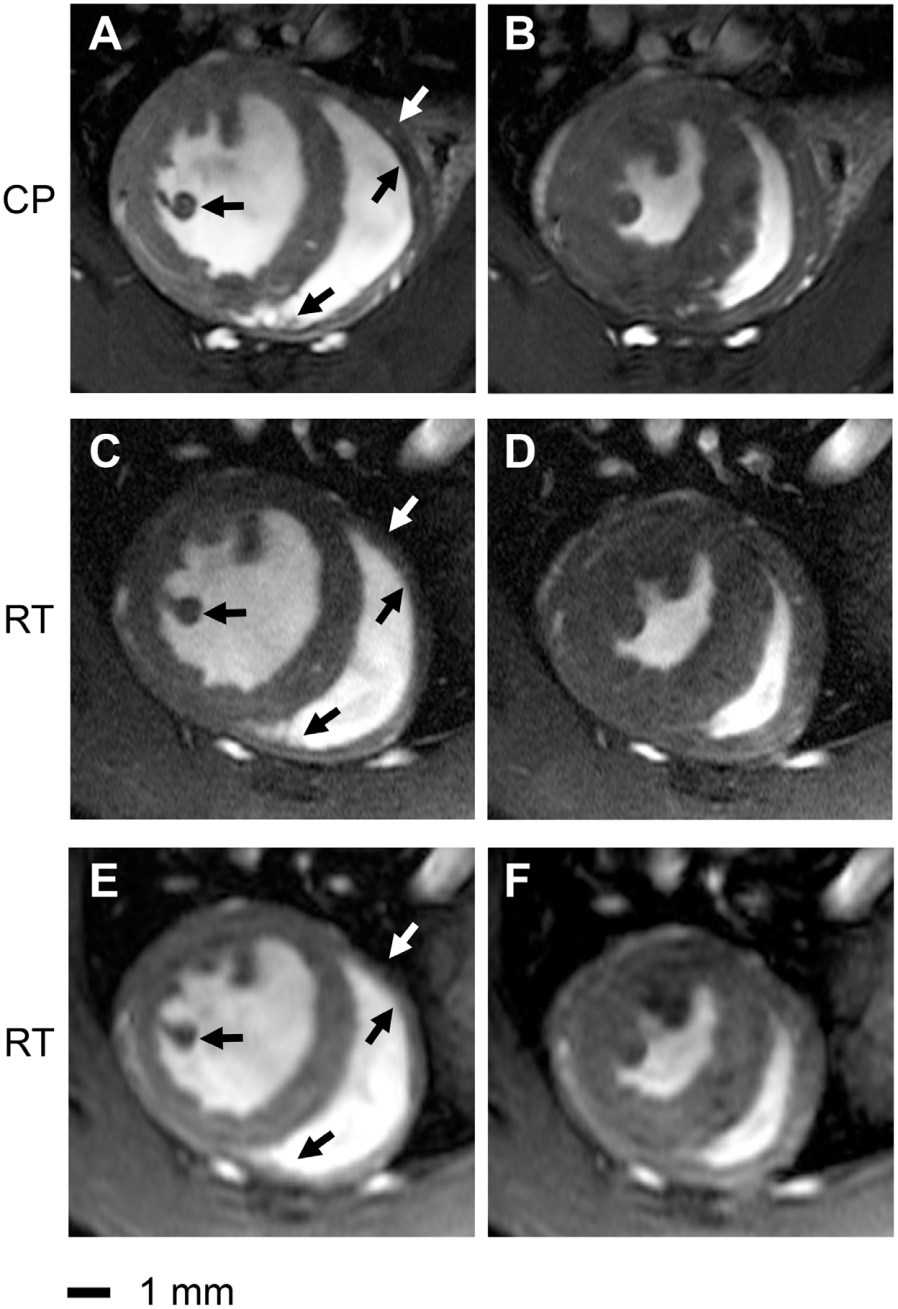
Comparison of end-diastole (left column) and end-systole (right column) short axis views acquired using a spatial resolution of 156×234×800 µm^3^ (a,b) and 69×115×800 µm^3^ (c,d,e,f). The depiction of anatomic details for left ventricular papillary muscles and right ventricular trabeculae is enhanced in the CryoProbe (CP) images (e,f) compared to the room temperature (RT) coil images (a,b,c,d) for both diastole and systole. Coronary arteries are more pronounced in the CryoProbe images. The lateral right endocardial boundary is better delineated in the CryoProbe images. The signal-to-noise ratio of the lower resolved images acquired with the RT-coil (47±9, a,b) is comparable to that of the higher resolved images acqured with the CryoProbe (54±7, e,f).

Cardiac cine imaging of the mouse heart in the short axis and four chamber view was feasible for both protocols and coil configurations. The high-resolution images derived from CryoProbe acquisitions revealed an enhanced anatomical detail and myocardial border sharpness versus the RT-coil acquisitions as illustrated in Figures 2 and 3. Figure S1 shows a short axis view CINE data set of 20 cardiac phases acquired with the CryoProbe. For the four chamber view (*Figure 2*), the CP image afforded an accurate delineation of the left ventricular outflow tract including the aortic valve and the aortic vessel wall. In comparison, identifying the closing of the aortic valve in the CINE images derived from RT acquistions is elusive. For the CP images the lateral right ventricle blood myocardium border was well defined especially at the level of the apex. In contrast, subtle anatomic structures were severly blurred by noise in the RT images: a close examination of the short axis views (*Figure 3*) revealed an enhanced depiction of anatomic details of the left ventricular papillary muscles and right ventricular trabeculae in the CP images compared to the RT images, for both diastole and systole. Coronary arteries were more pronounced in the CP-images. In particular, the lateral and inferior right endocardial boundary was better delineated in the CP images. Although the shape of the right ventricle differed between the prone position (RT coil) and supine position (CP) (*Figures 2* *+3*), the end-diastolic volumes (EDV) or end-systolic volumes (ESV) did not differ significantly: ΔEDV  = −2.13±11.37 µl, ΔESV  = 3.98±4.95 µl.

The SNR assessment showed that SNR derived from the lower resolved images acquired with the RT coil (SNR  = 47±9) was comparable to that obtained from the higher resolved images acquired with the CryoProbe (SNR  = 54±7). In contrast, the SNR of the higher resolved images acquired with the RT coil was only 15±2, and that of the lower resolved images acquired with the CP was 221±16.

**Figure 4:**
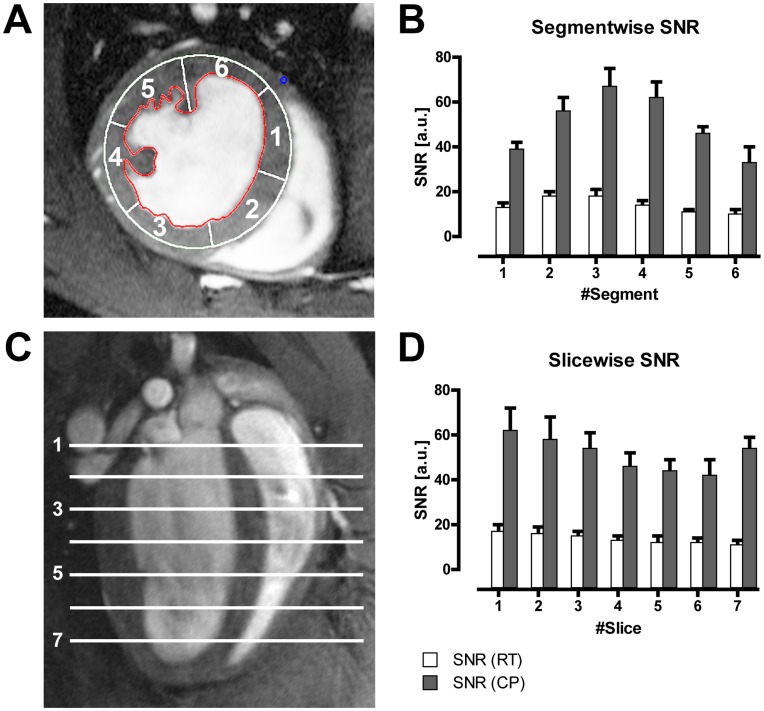
*a,b:* Bar plot of mean LV myocardium signal-to-noise ratio (SNR) (b) measured in images acquired with the CryoProbe (CP) or room temperature coil (RT) for all mice in the different segments of a six-segment model (a). The segments were numbered clockwise, starting at the inferoseptal segment. The region closest to the coil (segment 3) showed the highest SNR, and SNR decreased with distance from the coil. *c,d*: Bar plot of mean LV myocardium SNR (d) for all mice in the different slices (geometry shown in c). Slices were numbered from the basal slice to the apex. For the CryoProbe the apical slice showed an unexpected increase in SNR, in contrast to the decreasing trend from base to apex in the remaining 6 slices.

Both slicewise and segmented analysis of the mean SNR in the myocardium demonstrated superior SNR for the CP compared to the RT coil (*Figure 4*). The gain in SNR (CP vs RT) ranged from 3.0 to 5.0. Highest SNR gains were observed for the region located closest to the coil. The SNR gain was less pronounced for regions more distant to the CP/RT coil. Segmental SNR analysis resulted in: SNR_CP_/SNR_RT_  = 3.0, 3.2, 3.7, 4.3, 4.3, 3.2 for segments 1–6. The slice by slice SNR analysis revealed a minor reduction in the SNR gain from base to apex for both coils, with the exception of the apical slice in the CP images, where SNR increased again. For slices 1–6 a comparable mean SNR gain was observed: SNR_CP_/SNR_RT_  = 3.6±0.1. A peak SNR gain of 5.0 was achieved in the apical slice.

**Figure 5:**
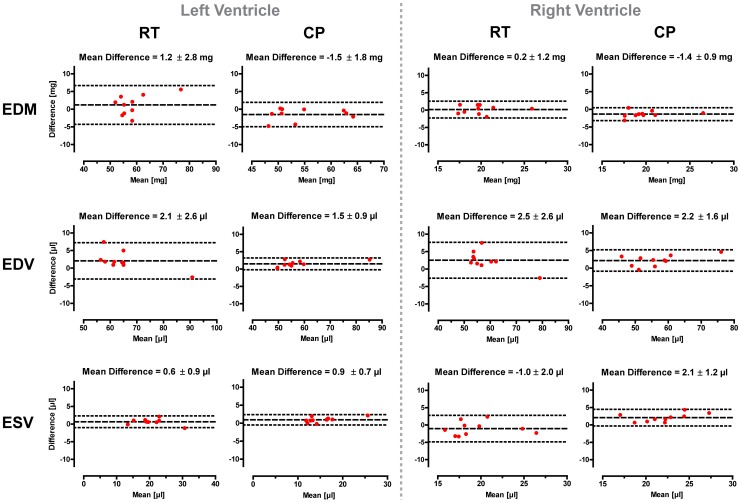
Bland-Altman plots of end-diastolic volumes (EDV), end-systolic volumes (ESV), and end-diastolic myocardial masses (EDM) for the left and right ventricle. For both ventricles and all three parameters the standard deviation of the differences was smaller for the CryoProbe than for the RT-coil. The LV-EDV and RV-ESV showed the largest impact, followed by the LV-EDM and the RV-EDM.

Bland-Altman analysis of intraobserver comparison for end-diastolic volumes (EDV), end-systolic volumes (ESV), and end-diastolic myocardial masses (EDM) for the left and right ventricle is shown in *Figure 5*. Standard deviations of the mean differences for EDV, ESV and masses were found to be smaller using the CryoProbe for both left and right ventricle. The LV-EDV and RV-ESV showed the largest impact, followed by the LV-EDM and the RV-EDM.

The analysis of the interobserver comparison yielded the following mean differences (± standard deviations, RT/CP): LV-EDV 0.0±1.4 µl/1.6±1.8 µl, RV-EDV−0.9±2.3 µl/−1.0±3.1 µl, LV-ESV−0.2±1.3 µl/1.1±0.6 µl, RV-ESV−1.7±1.0 µl/−1.6±1.0 µl, LV-EDM 1.6±3.7 mg/0.1±1.8 mg, RV-EDM 0.8±1.6 mg/0.7±1.0 mg. Using the CryoProbe standard deviations were smaller, except for the EDV.

**Figure 6:**
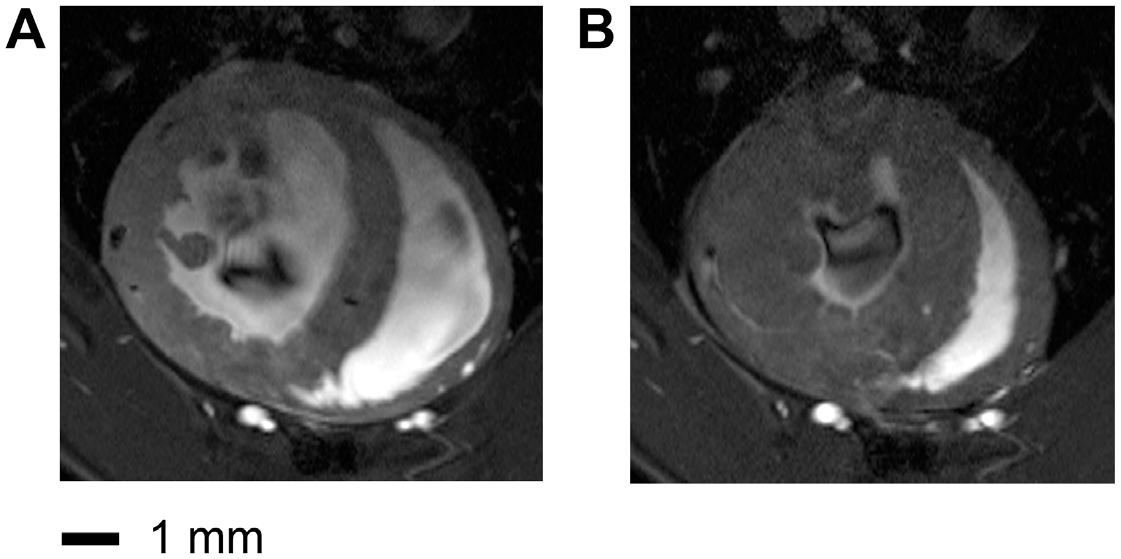
End-diastole (a) and end-systole (b) short axis views acquired using the CryoProbe together with a ultra-high spatial resolution of (43×138×300) µm^3^. Endo- and epicardial border delineation as well as the depiction of anatomic details of the papillary muscles and trabeculae are improved versus the high resolution CryoProbe images (Figure 3 e,f) due to the enhanced spatial resolution used in the ultra-high spatial resolution protocol.

The image quality of the CryoProbe ultra-high spatial resolution images (*Figure 6*) was even further enhanced compared to the high resolution images (*Figure 3*). Endo- and epicardial border delineation as well as the depiction of anatomic details of the papillary muscles and trabeculae were improved.

The partial volume effects for the very thin structure of the RV were estimated by calculating how many image voxels constitute the myocardium of the RV (mean volume 18.3 µl): 626, 2879, or 10266 voxels for the conventional, high, or ultra-high resolution protocols. With the improved spatial resolution afforded by the CP the RV wall (estimated wall thickness 0.40 mm) was covered by 3.5–5.8 pixels. For comparison, the RV was covered by only 1.7–2.6 pixels when using the RT coil with the lower resolution protocol. With the ultra-high spatial resolution protocol the RV was even covered by 2.9–9.3 pixels, which is beneficial for reducing partial volume effects.

## Discussion

In this study we demonstrated the feasibility of structural and functional CMR in mice employing a cryogenic RF surface coil. Image quality in terms of SNR and depiction of anatomic details was found to be superior using the CP compared to the standard cardiac four channel room temperature surface coil in conjunction with the volume resonator. Intraobserver variability improved for the assessment of cardiac volumes and masses and interobserver variability for ESV and EDM.

The B_1_^+^ decrease from anterior to inferior in the short axis view is approximately linear and less pronounced than that reported for phantom experiments by Baltes *et al* [16]. This may partially be due to the use of the double-oblique imaging slices, leading to the short axis view and four chamber view showing less of a B_1_^+^-gradient than one would expect in an axial slice and more than one would expect in a coronal slice.

B_1_^+^ values in the heart were found to be above 100%. This can be explained with the fact that the transmitter adjustment slice was placed in a strictly coronal orientation using a strictly axial reference image, while the heart orientation is oblique. Another limitation was that an entire slice was used for transmitter adjustment rather that a selected volume. Further optimization of the positioning of the RF-power adjustment slice and ideally a spatial limitation to a selected volume (through a modification of the adjustment procedure) might help to reduce this deviance and to further improve SNR and blood-myocardium contrast. Nevertheless, the B_1_ characteristics afforded excellent image quality and facilitated cardiac chamber quantification.

The observed differences beween the shape of the right ventricle in the RT coil images and CP images can be explained with the different positioning of the animal, i.e. in the prone position for the RT coil and in supine position for the CP. Besides the natural prone position, also the supine position has been used for CMR studies of mice (e.g. [5], [9], [22]). An effect of the positioning on the functional parameters cannot be ruled out completely, but is unlikely since the end-diastolic and end-systolic volumes of the RV did not differ markedly in our study.

The demonstrated SNR improvements for images acquired with the CryoProbe compared to the RT-coil set-up is consistent with the published findings for MRI of the brain at the same field strength of 9.4 Tesla and with similar RF hardware [16]. For cardiac MRI the achievable SNR gain reported here exceeds that reported for brain (heart: 3.0 to 5.0, brain: 2.2 to 3.0). One possible reason for the differences in SNR gain in the present study is a different performance of the room-temperature coil that was used as reference. However differences in the CryoProbes cannot be excluded, notwithstanding same design.

The spatial variation in SNR across the myocardial segments of a short axis view can be explained with the proximity to the surface coil. A decrease in signal-amplitude with increasing distance is inherent to surface coils. Here, the surface coil characteristics explain that the region closest to the coil showed the highest SNR, and SNR clearly decreased with the distance from the coil.

A trend towards a SNR reduction along the base to apex line was observed, with the exception of the apical slice (CP only). The unexpected higher SNR at the apical slice observed for the CryoProbe was not found for the RT images. Hence partial volume effects, i.e. blood contaminations, can be excluded as a reason for this SNR increase. Moreover, the same SNR increase in slice 7 was found also for a region of interest in the skeletal muscle (located within the slice). This phenomenon is therefore not likely to have physiological causes, but conceivably may be caused by the two-element surface coil characteristics.

The achieved gain in SNR by use of the CryoProbe facilitated higher spatial resolution CMR, while maintaining an SNR comparable to the lower resolution RT-coil images. While the high resolution protocol also yielded a better depiction of anatomical details in RT-coil images when compared with the respective lower resolved images, this came at the cost of significantly reduced SNR and poorer delineation of myocardial boundaries, particularly for the RV. The subjective criteria of enhanced image quality evident in the high resolution CP images was underpinned objectively by the results of the quantitative assessment of cardiac function and morphology. These encouraging results further support our subjective impression of excellent image quality in the high-resolution CryoProbe images and suggest that CMR-based assessment of cardiac function and morphology in mice for both left and right ventricle could benefit from the use of a cryogencally cooled RF coil.Furthermore, the high SNR of the CryoProbe allowed for pushing the limits of spatial resolution for *in-vivo* CMR of mice using a horizontal bore MR system even further to 43×138×300 µm, resulting in superb delineation of endo- and epicardial borders, papillary muscles and trabeculae. Images of this quality for some selected slices of particular interest could complement whole heart coverage by means of the high resolution protocol. This work demonstrates the SNR benefits of the CryoProbe approach in healthy mice. This mandatory precursor was essential before extra variances due to gender and/or pathophysiological conditions are introduced. It is to be expected that the SNR gain shown here will not be altered significantly when moving towards pathological conditions. Realizing this opportunity we anticipate to put the proposed high spatial resolution CryoProbe approach to use for rapid characterization and phenotyping of mouse models of cardiovascular diseases.

In conclusion, cardiac morphology, cardiac chamber quantification and cardiac function assessement using a cryogenically cooled RF probe is feasible and affords SNR gains in the range of 3.0 to 5.0 compared to a conventional room temperature cardiac RF coil set-up. This SNR gain could potentially be invested in shortening the acquisition time. Yet, more relevant to CMR, the increased SNR enables a higher spatial resolution. This appears to facilitate a more accurate quantitative analysis, due to reduced intraobserver variability. In summary, the use of a cryogenically cooled RF probe represents a valuable means of enhancing the image quality in CMR of mice. This improvement holds the promise to be beneficial for cardiac MR research explorations into a variety of mouse models of cardiovascular diseases including the characterization of progression and regression of hypertension induced myocardial hypertrophy and identification of sex specific effects of cardiac damage and cardiac (dys) function.

## Supporting Information

Figure S1 **Short axis view CINE data set of 20 cardiac phases of the mouse heart acquired at 9.4 T using the CryoProbe together with the high spatial resolution protocol.** End-diastolic (frame 1) and end-systolic (frame 11) cardiac phases are marked by a red and blue outline respectively.
(TIF)
