# Supplementary figures and images for: Functional and Morphological Cardiac Magnetic Resonance Imaging of Mice Using a Cryogenic Quadrature Radiofrequency Coil
Source: PLoS One. 2012 Aug 1;7(8):e42383. doi: 10.1371/journal.pone.0042383 (PMC3411643; doi:10.1371/journal.pone.0042383)

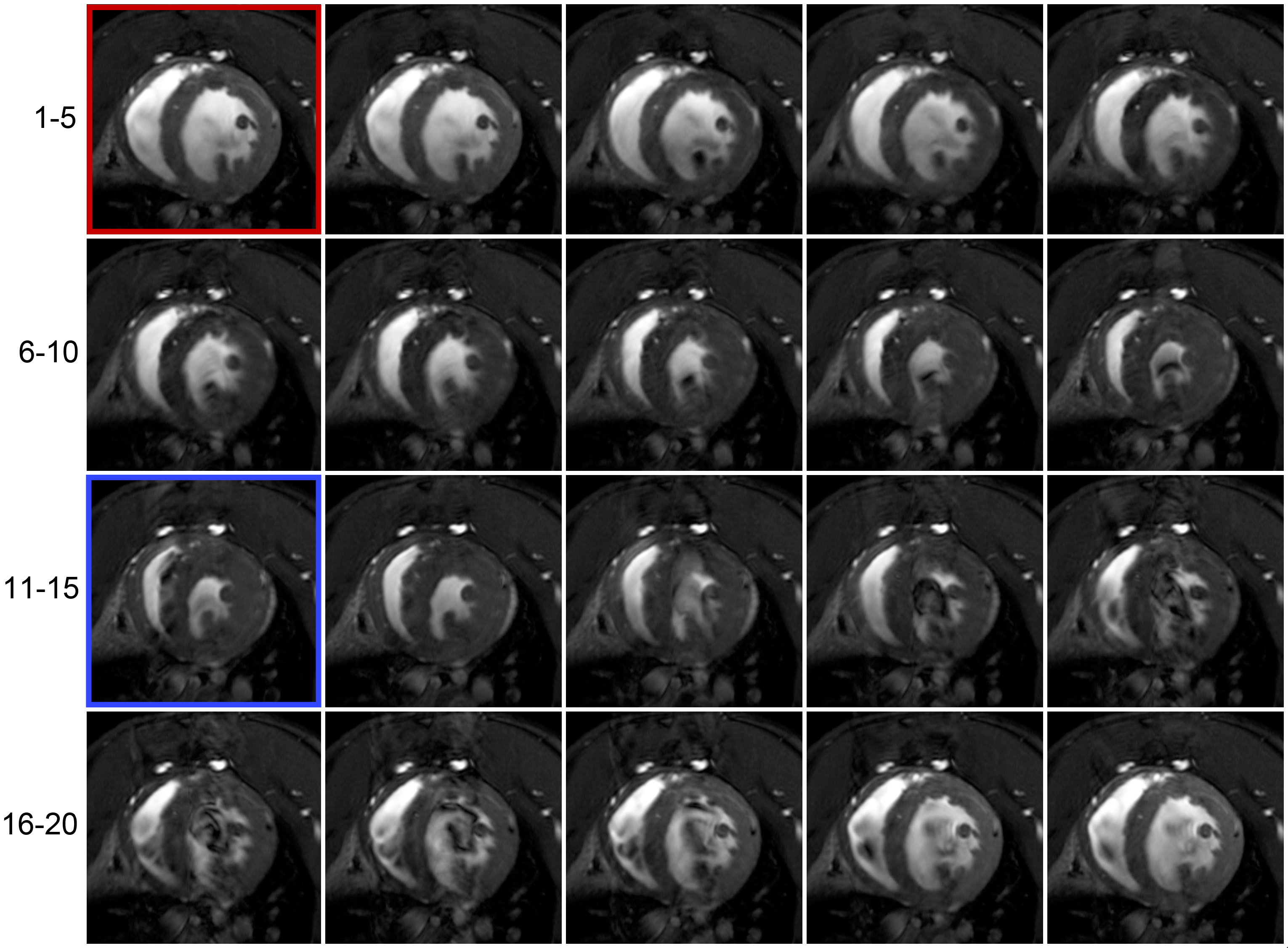

Supplement: Figure S1 — Short axis view CINE data set of 20 cardiac phases of the mouse heart acquired at 9.4 T using the CryoProbe together with the high spatial resolution protocol. End-diastolic (frame 1) and end-systolic (frame 11) cardiac phases are marked by a red and blue outline respectively. (TIF) [file pone.0042383.s001.tif]
